# Perspective: 50 years of plant chromosome biology

**DOI:** 10.1093/plphys/kiaa108

**Published:** 2021-02-19

**Authors:** Richard B Flavell

**Affiliations:** International Wheat Yield Partnership, 1500 Research Parkway, College Station, TX 77843, USA

## Abstract

The past 50 years has been the greatest era of plant science discovery, and most of the discoveries have emerged from or been facilitated by our knowledge of plant chromosomes. At last we have descriptive and mechanistic outlines of the information in chromosomes that programs plant life. We had almost no such information 50 years ago when few had isolated DNA from any plant species. The important features of genes have been revealed through whole genome comparative genomics and testing of variants using transgenesis. Progress has been enabled by the development of technologies that had to be invented and then become widely available. Arabidopsis (*Arabidopsis thaliana*) and rice (*Oryza sativa*) have played extraordinary roles as model species. Unexpected evolutionary dramas were uncovered when learning that chromosomes have to manage constantly the vast numbers of potentially mutagenic families of transposons and other repeated sequences. The chromatin-based transcriptional and epigenetic mechanisms that co-evolved to manage the evolutionary drama as well as gene expression and 3-D nuclear architecture have been elucidated these past 20 years. This perspective traces some of the major developments with which I have become particularly familiar while seeking ways to improve crop plants. I draw some conclusions from this look-back over 50 years during which the scientific community has (i) exposed how chromosomes guard, readout, control, recombine, and transmit information that programs plant species, large and small, weed and crop, and (ii) modified the information in chromosomes for the purposes of genetic, physiological, and developmental analyses and plant improvement.

## Introduction

Fifty years is a long time, but in scientific discovery and application it can be inadequately short. Choosing a time period of 50 years for this “Perspective” is somewhat arbitrary but it is long enough to be meaningful to provide commentary on the evolution of scientific progress. It also happens to be the period that I have spent in plant chromosomal science (1969–present). I entered plant chromosome research from the world of microbial and fungal genetics by joining the Cytogenetics Department of the Plant Breeding Institute at Cambridge, UK. That set me on the path of wanting to know much more about plant chromosomes and what we need to know about them to benefit plant improvement in new ways. In the mid-1970s, recombinant DNA technologies had emerged and genes introduced into bacteria and yeast with the new DNA being incorporated into their chromosomes. This enabled the effects and functions of the introduced pieces of DNA to be assessed in vivo and mutations to be complemented. If this was the new synthetic genomics and means of making targeted genetic changes in bacteria and yeast, why not in plants, including crops, to solve problems in agriculture? In 1977, the phi X174 genome was sequenced. If a phage genome, why not a plant chromosome? The vision was very appealing. The 50 years has therefore been a quest to seek, interpret, and change information that programs plant life and agriculture.

Chromosomes not only contain the information for plant life but also have been selected over millions of years to transmit, sustain, and manage it. Genetic information is precious. It must be replicated every cell generation and between plant generations but not be allowed to accumulate harmful mistakes. Yet variation essential for evolution must be recombined in meiosis. The genetic information is used only at certain times or in some cells and so its use needs to be regulated during development and in response to environments, while going through programmed condensation and decondensation cycles. Also, it is the sets of homologous chromosomes within each species that hold the variation upon which evolution and plant breeding depend. Knowing this, variation is essential for building new platforms for plant improvement. From the last 50 years of global investment, we are now able to appreciate chromosomes as integrated functional information entities. We have also unleashed molecular genetics and learnt to change chromosome information to alter the properties of plants, including crop species. In the process of generating so much understanding we have created different ways to think about plants, their evolutionary past, and their potential future. The intellectual journey has been rich and rewarding.

## How has the story progressed over the 50 years?

Plant genetics and associated chromosome biology were relatively uninspiring in the 1960s compared with what was happening in fungal, bacterial/phage, and drosophila genetics and when the genetic code was pronounced to be universal after being found to be the same in bacteriophage, tobacco mosaic virus, and humans. Plant science lacked the equivalent intellectual appeal and experimental penetration. The visual appeal of colorful flowers did not win over the sharp mind of the entrepreneurial researcher. Who wanted to deliver more description when it was possible to discover universal mechanisms of life?

**Figure 1:**
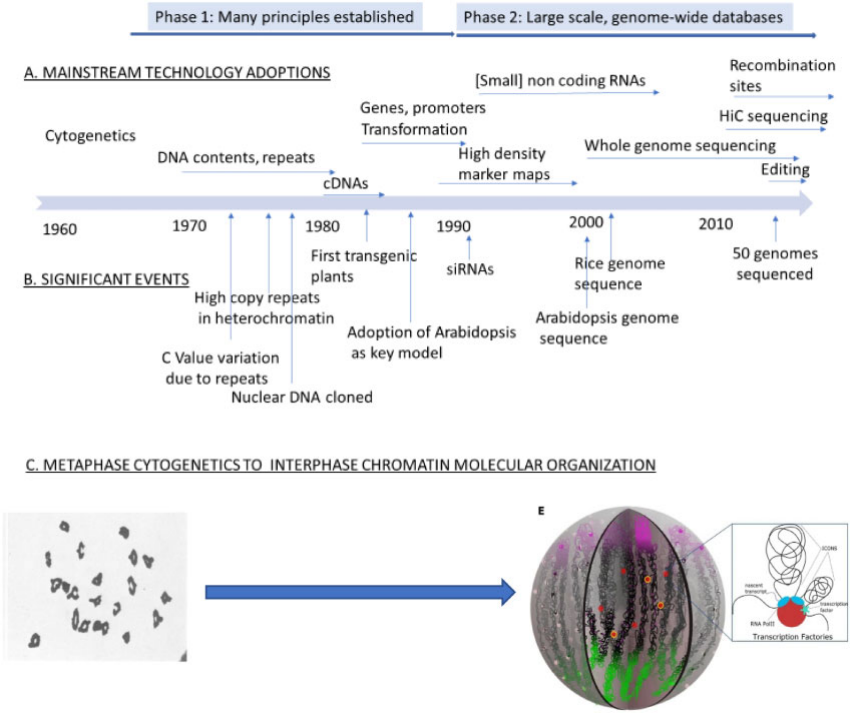
Progress of plant chromosome research over 50 years. A, A timeline showing when significant technologies were first adopted by frontrunners in the plant science community. The arrows underneath each indicate that use of the technologies continues forward to the present. B, The timing of some significant landmarks in research progress. C, Symbolization of the major developments in chromosome research over the 50 years. In the early years studies were focused mainly on metaphase chromosomes because little could be discerned in interphase chromosomes, except heterochromatin. Now, details of interphase chromatin are being described at the subgene level as a result of full analyses of genes and genomes and application of HiC and related technologies. Left hand picture: The 21 wheat meiotic bivalents photographed in the group of cytogeneticists that I joined in 1969 (Bayliss and Riley, 1972). Right hand picture: Model of wheat interphase chromosome organization in which segments are brought together into transcription factories, taken from Concia et al. (2020) (see the “Chromosomes have 3-D order and links to one another in nuclei” section).

Nevertheless, plant science did grow from the 1960s based on the discoveries being made in non-plant species. During the first 25 years (say, 1965–1990; see Figure 1) most of the currently known principles of chromosome biology were established in plants, including knowledge of repeated sequences and their contribution to genome size, knowledge of DNA variation, gene structure and promoters/terminators, introns, DNA replication, basic chromatin structure, epigenetics, and gene control as well as establishing the means of adding new genes by transformation. All these were large achievements for plant science but for the most part illustrated that what had been discovered in animals, bacteria, and fungi applied to plants too, endorsing remarkable uniformity across the living world—a very important conclusion at the time. The second 25 years (say, 1990–2015; see Figure 1) addressed scale—how to gain knowledge about large numbers of specific genes, gene expression networks, molecular genetic maps, and whole genome sequences and how to create the big data packages and their algorithm-based analyses to achieve better understanding of the molecular genetics of whole organisms and variation within and between species via comparative genomics. In addition, discoveries on the role of histone variation, small RNAs, and longer non-coding RNAs in regulating chromatin structure and hence gene/chromosome activity have provided much new insight into how repeated sequences, transposons, and genes are kept under control. The resulting vistas of chromatin structure along whole chromosomes combined with the knowledge of the epigenetic control systems are providing a much stronger basis for understanding the molecular features of chromosomes that have evolved to sustain the diversity of plant species. Important knowledge on recombination was also generated.

## Repeated sequences comprise much of the DNA in chromosomes

The group of chromosome experts that I joined in 1969 scrutinized metaphase chromosomes and their meiotic behavior as cytogeneticists (Figure 1, C). In those days, cataloguing the DNA contents of plant species became a significant activity and continues to this day; over 12,000 plant species have now been so defined. There is a 3,000-fold variation in DNA contents across the plant kingdom (Bennett and Leitch, 2005). After demonstrations that the volume of the metaphase chromosome is proportional to its DNA content, the major question was why are there such large differences in DNA content between species? This *C*-value paradox, namely the large variation in DNA content between species and where DNA amounts do not relate to organismal complexity, was actively debated for many years (Cavalier-Smith, 2005; Freeling et al., 2015).

The late 1960s and 1970s were significant times because they involved a switch from cytological approaches to ones based on isolated DNA. I moved into isolating DNA to find out things that were buried in metaphase chromosomes and not visible to the cytogeneticist. I adopted the powerful set of techniques based on DNA renaturation kinetics devised and championed by Britten and Kohne (1968) to reveal features of cereal chromosomal DNAs (Smith and Flavell, 1975; Flavell and Smith, 1976). Isolated DNA was sheared by sonication to small fragments (300–600 base pairs), denatured, and allowed to renature under defined conditions in which identical or very similar DNA sequences would undergo Watson–Crick base pairing. Under these conditions, the rate of renaturation is related to the concentration of complementary DNA sequences in the reaction; copies present in high concentration renature faster than sequences present rarely. The approach enabled the numbers of nearly identical copies within a family to be estimated and to some extent the families distinguished. An early renaturation curve for wheat (*Triticum aestivum*) DNA illustrated that some 80% of the total DNA renatures with the kinetics of repeated sequences (Smith and Flavell, 1975; Flavell and Smith, 1976). In fact, it was closer to 100% when diverged repeats that behaved as non-repeats were taken into account. Thus, the genes that behaved as single Mendelian units were likely to occupy less than 1% of the total DNA. Similar surveys of many species led to the provocative conclusions that (i) animal and plant genomes have high concentrations of families of identical or nearly identical sequences and (ii) that most of the differences in DNA content between species is due to the accumulation of different amounts of repeated DNA, unless polyploidy was the reason (Flavell et al., 1974).

Repeated sequences feature as a theme in this perspective because they dominate chromosomal DNA, particularly for the cereal species that I started with 50 years ago. As this perspective emphasizes, repeated sequences are now known to play a significant part in regulating minimal cell division rates and life cycle times as well as contributing to local chromatin architecture, transcription, recombination, and 3-D nuclear structure. Their ability to program instability into chromosomes because of their potential for transcription, transposition, and sequence-based recombination was recognized in the early part of this 50 year period while only in the last 20 years have the RNA-directed mechanisms that keep them quiescent been elucidated. Indeed, we knew nothing about these mechanisms in the 1970s. They have now contributed much to the bringing together of the various aspects of chromosomal biology, including plant transformation, into a unifying, three dimensional, local chromatin-based vision, as this perspective reveals.

## Repeated sequence comings and goings generate much diversity between species

Comparative interspecies renaturation studies, especially our own on cereal species (Smith et al., 1976; Rimpau et al., 1978) led to the conclusions that during species divergence new families of sequences were generated and fixed and family members became interspersed across the genome. Others became lost. Thus, the DNA in chromosomes is dynamic and repeated sequences turn over during evolution (Flavell, 1982). The sizes of the pieces of DNA that were amplified and inserted in these cereal genomes were short—around 1,000 bp or less on average. Thus, we concluded that these genomes were built almost entirely from millions of short sequences and different high copy repeat units were interspersed with each other as well as with low/single copy sequences, except for the arrays of tandem repeat units typical of the heterochromatic C bands (Smith et al., 1976; Rimpau et al., 1978).

The dispersion of near identical copies around the genome must have come about by movement of sequences, by transposition. Later studies of cloned repeat units in the wheat genome confirmed the diverse, interspersed organization of specific repeat family members (Bedbrook et al., 1980b). It emerged subsequently that two broad categories of transposable elements were common. Elements in one class move by excision from the genome to be reinserted elsewhere, usually to a nearby site, by “replicative transposition.” Elements in the other, more frequently observed, class gain new positions in the genome by reverse transcription of their RNA transcripts into DNA followed by reinsertion of DNA copies into the genome, creating identical long terminal repeats (LTRs). Today, most of the families of transposable elements in many plant genomes have been thoroughly classified, sequenced, and their evolution traced by a variety of research groups (e.g. Han et al., 2013; Bennetzen and Wang, 2014)*.* All these features of sequence organization are now obvious in fully sequenced genomes (see the “Whole chromosome DNA sequencing reveals organizational order” section) and nested transposons of different types are now routinely found in the repeated regions of plant chromosomes, e.g. maize (*Zea mays*; San Miguel et al., 1996), as predicted in the models formulated in the 1970s (Smith et al., 1976; Rimpau et al., 1978; Bedbrook et al 1980b).

The most highly repeated sequences in these cereal genomes could be isolated, tritium labeled, and hybridized to denatured DNA in metaphase and interphase nuclei fixed on microscope slides. They commonly hybridized preferentially to the constitutive heterochromatic bands around telomeres and/or centromeres which were inferred to contain high concentrations of specific repeats (Jones and Flavell, 1982a). There were substantial differences between closely related species (Jones and Flavell, 1982b). When in the late 1970s/early 1980s we had cloned individual members of repeated sequence families using recombinant DNA techniques, similar experiments endorsed the same conclusions (Bedbrook et al., 1980a). These studies thus linked DNA sequences to visible metaphase chromosome architecture and, as described in the “Chromosomes have 3-D order and links to one another in nuclei” section, to nuclear architecture.

From these initial studies, models were generated for the genomes of wheat, rye (*Secale cereale*), barley (*Hordeum vulgare*), and oats (*Avena sativa*; Smith et al.,1976; Rimpau et al., 1978; Rimpau et al., 1980) based on how members of the repeated DNA families diverge and new families arise by amplification of recombinant transposable elements created from existing ones, to generate the observed genome turnover. Many recombinant elements have been described in barley, for example (Vicient et al., 2005) that add much to our models of the 1970s, including the active element BARE 2 (barley retroelement 2) that appears to be a recombinant between an older BARE 1 element in the barley genome and one similar to the Wis-2-1A (wheat insertion sequence-2-1A) element we characterized in wheat (Murphy et al., 1992). New elements can also arise from horizontal transfer from other plant species (El Baidouri et al., 2014).

How amplification and deletion have played major roles in genome evolution is now fully appreciated. For example, the genome size of rice has doubled in the last 5 million years, largely due to retrotransposon bursts (Vitte et al., 2007). Transposable elements in the ancestors of maize have undergone massive bursts lasting thousands or millions of years. However, because modeling studies suggest that waves would be silenced more rapidly, these large bursts may have consisted of many smaller and shorter bursts (Roessler et al., 2018). The wheat genome has been shaped by successive waves of amplification during evolution (Dai et al., 2018) followed by periods of silence. The more rapid the burst the quicker the silencing was established. This might be expected for species to survive. Arabidopsis (*Arabidopsis thaliana*) has evolved from an ancestral form with a much larger genome by loss of DNA via large-scale rearrangements but particularly by hundreds of thousands of small deletions throughout its genome, presumably under selection to have a shorter life cycle (Hu et al., 2011). How selective forces act in plants to counteract transposon bursts using purpose-designed silencing systems are actively being studied to understand more about the huge changes that chromosomes undergo (see the “Transcripts and small RNAs regulate chromosome activity through chromatin/epigenetic modifications” section).

Early DNA sequencing showed that members of some repeated sequence families are more identical than would be expected for amplified sequences evolving independently of one another. Homogenization of repeats was clearly occurring. This was likely to be by gene conversion and concerted evolution-like processes which can result in reductions in mutational variants in a population and homogeneity within an array or family (Dover and Flavell, 1984). Ribosomal RNA genes were an early, much studied example of this in plants and other organisms. Dover championed the proposal that such homogenization mechanisms would lead to new phenotypes and species irrespective of or in concert with Darwinian selection. This was never fully accepted. Studies on centromeres provided additional dimensions to such discussions. It is now known that the repeats that are concentrated in centromeres bind the centromere-specific histone H3 protein (CEN H3) to coordinate the interaction of chromosomes with tubulin proteins, the kinetochore, that ensures faithful chromosome segregation during mitosis and meiosis (Simon et al., 2015). Thus, selection is likely to occur for similarity, if not identity, between centromere repeats on all chromosomes within a species. Selection is also likely to occur on repeats close to genes that affect gene activity (Hirsch and Springer, 2017).

The dynamic nature of repeats in plant genomes raised many questions central to chromosome biology, evolution, and gene function that set major challenges for the decades to come. Where much of a genome was comprised of repeated families of DNA whose members could move around the genome, the potential genetic load through disruptive mutations would be massive, unless the movement was kept to essentially zero per generation. Therefore, one of the biggest challenges was to discover how these repeated elements were kept silent. Another was to determine what differences they made to a plant species, given their diversity, even among closely related species. How have all the repeats co-evolved with the chromosomal functions on which plant species depend? All these issues have become discussed much more recently as genomes have been sequenced and the domination of repeats and LTR transposons in genomes fully recognized.

## DNA contents affect rates of plant development and minimum generation times

While the early studies on repeated DNA sequences revolutionized our view about the DNA in chromosomes, they provided little knowledge about why families of repeated sequences are there and any functions they may have, apart from the genetic observations of McClintock (1951) which were clearly seminal in showing how transposons near genes can influence their activity. Some of us respected the selfish DNA hypotheses (Orgel and Crick, 1980) based on the notion that transposons arose and replicated in host chromosomes for their own ends but believed that it was inconceivable that selection would not have put such DNAs to good use in a variety of ways. Some at least would surely have been a resource for useful evolutionary novelty, however, they arose. Debates have continued to rage over the years about the *C*-value paradox and whether at least some of the DNA is simply junk DNA without any (sequence or position-based) function. I have subscribed to the view that we should recognize that (i) functions can be assessed at many different levels from chromosomal process to population, (ii) functions may not be based only on sequence informational criteria but also on nucleotypes-total DNA amounts and that (iii) traits may arise neutrally initially but can be preserved by purifying selection later, for example by individual members of a repeat family becoming genetically functional when close to a gene (Doolittle, 2013). Most but not all members of families of repeats are now viewed as examples of parasites, or one-time parasites, to which the host species has adapted and subsequently exploited for providing novel sources of genetic variation (Freeling et al., 2015). As more details of chromosome functions and controls are uncovered, more roles for repeated DNA are becoming defined (Freeling et al., 2015; Makarevitch et al., 2015; Hirsch and Springer, 2017).

During the 1970s and 1980s, my colleague Michael Bennett and others shed some light on possible functions or effects of repeated DNA and hence genome size by linking DNA contents to cell size, cell cycle time, and plant development (reviewed in Bennett and Leitch, 2005). Total nuclear DNA content (“nucleotype”) correlated with cell size and the volume of certain organs, such as pollen, across the plant kingdom. DNA content also correlated with minimum cell cycle time for diploids. In general, more DNA slows down cell cycles and increases minimal developmental rates. If DNA contents surpass a certain value the plants become obligate perennials. Amplification and deletion of highly repeated DNA can therefore provide a rheostat to adjust developmental rates, presumably in conjunction with the properties of specific, expressed alleles in the genotype and the degree of heterosis. The DNA content, the so-called “nucleotype,” therefore influences properties of the species irrespective of the particular sequences in the chromosomes and is under selection. This was clearly an important addition to the concepts of Mendelian genetics and specific gene function and an important background for all considerations of chromosomes and genomics.

Over the years numerous examples of the “nucleotype” correlating with plant phenotypes have been published. For example, there is a negative correlation between nuclear DNA content and flower size (petal limb length, petal claw length, and calyx diameter) in *Silene* (*Silene vulgaris*) both in wild accessions and in response to artificial selection (Meagher and Costich, 1994). Also, selection for earlier flowering in maize resulted in a correlated reduction in DNA content (Bilinski et al., 2018). The major losses in DNA during the evolution of Arabidopsis and rice are consistent with this concept.

## Adoption of model species revolutionized plant science

Plant species with large chromosomes that were ideal for studies in the era of cytology and on families of repeats were far from ideal for genetics and to study genes at the DNA level. Their genomes were too big, the plants were often too large physically and their life cycles too long. In the early 1980s, it was obvious to many that we desperately needed a species that would enable the unlocking of the molecular biology of plant chromosomes on a vast scale, to provide detail on all the genes, their roles alone and in combination, and to launch understanding of the detail of the genetic basis of biochemistry, physiology, and growth. In short, to do what was happening in *Escherichia coli*, *Bacillus subtilis*, *Saccharomyces cerevisiae*, *Caenorhabditis elegans*, *Homo sapiens*, and other model species. Plants should not be left behind! Thus, the scientific communities debated what could be done. A species with a small genome, good diploid genetics, a short generation time, and that could be transformed was essential. Tobacco (*Nicotiana tabacum*) and petunia (*Petunia hybrida*) held promise in some debates because their tissue culture and regeneration to whole plants was well worked out, but Arabidopsis won out for a variety of reasons, nicely documented by Somerville and Koornneef (2002). By the end of the 1980s several groups in Europe, including ours at the John Innes Centre, UK, had initiated major programs of research on Arabidopsis. The need to formulate an international effort strengthened by the end of the 1980s, and pressure was put on the major funding bodies, especially in the USA and Europe, to adopt Arabidopsis. Adoption of a non-crop model was very controversial. However, the Multinational Co-ordinated *A. thaliana* Genome Project was born (Meyerowitz et al., 1991) with a small group of us representing USA, Europe, Asia, and Australia as the first steering committee.

While Arabidopsis did and does still today attract more researchers than any other species, especially in Europe and in the USA, rice became a model for discovery and application and complemented Arabidopsis nicely with its small genome and facile regeneration from cell culture and because it is a monocot. Today the literature describing the knowledge and tools for rice research is immense (Juanillas et al., 2019; Oryzabase, 2000). The need for another monocot model gave rise, especially in the USA, to the adoption of *Brachypodium distachyon*, a wild grass species, because of its small genome, rapid life cycle, and now, easy tissue culture and rapid plant regeneration. Much useful genetics and genomics has been achieved using it (Catalan et al., 2014). The arguments for adopting model plant species have been completely justified. They have dominated the discoveries. It is hard to imagine what would have happened to plant science if researchers and funders had not adopted model species. Much hard work had to be put into working out easy procedures and generating genomic resources for these model species. Tremendous thanks are due to the pioneers. They changed plant science for ever.

## Whole chromosome annotated DNA sequences unleashed massive understanding of chromosome structure

The sequencing of the yeast, fly, *C. elegans* genomes and the commitment to sequence the human genome were inspirations to tackle a plant genome. It had to be done. Arabidopsis was an obvious candidate. This was achieved in 2000 (Arabidopsis Genome Initiative, 2000). The assembly of 125 Megabases, built upon years of learning into making genomic libraries, sequencing, assembly of non-ambiguous contigs, and using much enhanced computing power, was clearly another landmark in plant biology. It was the product of a multinational program in which different groups sequenced different chromosomes, building on sequencing projects started by the EU in 1991. It was a triumph of international cooperation, although it was far from trouble free. Since then the 1001 Arabidopsis Genomes Initiative launched in 2008 and completed in 2016 (Alonso-Blanco et al., 2016) with the publication of 1,135 genomes is an even larger achievement and resource, especially given the available phenotypic information of the accessions, their methylomes, and the volume of research that has been built upon exploring the variation in these genomes.

Although I had been one of those who had pushed in the late 1980s for the achievement of a public Arabidopsis genome sequence, it was nowhere near complete when I arrived in the USA from the UK John Innes Centre in 1998 to be CSO of the plant genomics company, Ceres, Inc. I made this career move to be able to embark on much higher throughput studies into the complement of genes in plants, something more aligned with the needs of plant breeding. There was debate in the company initially about whether a more focused, higher throughput approach could complete the genome sequence faster. Monsanto, it was rumoured, was also seeking to create an Arabidopsis genome sequence. In the end, Ceres entered plant genomics by making libraries of full length cDNAs from Arabidopsis, maize, soybean (Glycine max), and some other species, followed by sequencing approaches that were very high throughput for that time. The approach enabled us to get a first view of tens of thousands of gene and protein sequences in each species, as well as the associated transcription start and termination sites, that were later published (Alexandrov et al., 2006, 2008). From deep sequencing of mRNAs we discovered multiple start sites for many genes, suggesting that genes are used differently in different cell types. Later, analyses across thousands of promoters enabled common structures of transcription start sites to be described (Troukhan et al., 2009).

When the Arabidopsis chromosome sequences were first published we knew that the annotations that defined genes (gene models), their transcription starts and stops, and where introns would be spliced out were relatively poor. Our full length, sequenced cDNAs showed this. We therefore offered 5,000 of them to the TIGR (The Institute for Genome Research) team concerned with annotating the public Arabidopsis genome effort. These full-length Arabidopsis cDNA sequences generated in Ceres, Inc. were used to greatly improve the precise start, stop, and intron processing annotations and at the same time improve the algorithms of the automated gene-calling software (Haas et al, 2002; Alexandrov et al., 2006). Our early analyses of the Arabidopsis genome using cDNAs discovered 148 new gene loci and 85 antisense transcripts to known genes, with alternative splicing occurring in some 7% of the genes (Alexandrov et al., 2006). Use of our full length cDNAs from other species helped considerably too, because coding sequences/protein sequences are usually adequately conserved and so can be identified across genomes. When we published our 31,000 non-redundant maizec DNA sequences these also aided annotation of the maize and other monocot genome sequences (Alexandrov et al., 2008).

More-or-less complete japonica and indica rice genome sequences were published in 2002 (Goff, 2002; Yu, 2002). Polished full genome sequences were released a few years later (International Rice Genome Sequencing project, 2005; Yu et al., 2005). These achievements also unleashed huge amounts of knowledge on genes, gene–trait links, cataloguing of genetic variation, molecular marker maps, and tools used heavily for comparative genomics across monocots (e.g. Juanillas et al., 2019; Oryzabase, 2000). In a recent landmark study of 3,000 rice genomes (Wang et al., 2018), large numbers of genomic polymorphisms created by transposition have been defined but most are present at low frequency and probably arose recently in agriculture (Carpentier et al., 2019).

Since the publications of the Arabidopsis and rice genomes, some 600 complete genome assemblies have been published (Kersey, 2019), having increased from 50 in 2013 when most sequenced were between 300 and 900 Mbp, following on from the 125 Mbp of Arabidopsis in 2000 (Michael and Jackson, 2013). These are found in the international genome databases. Genome sequences of several monocot species with larger genomes have been published over the past 5–10 years. For example, the maize genome sequence was published in 2009 (Schnable et al., 2009) but by this year some 40 genome assemblies are expected to be in the public domain. These numbers record the explosion in plant chromosome research for crop improvement. Many genome sequences revealing the methylated cytosine bases have also been published.

For me, a landmark was the completion of the hexaploid wheat genome sequence, with its 16.5 billion nucleotides, by an international consortium, with initial publication in 2014 based on a strategy of isolating individual chromosome arms by flow cytometry before sequencing and assembly (International Wheat Genome Sequencing Consortium [IWGSC, 2014] and additional improvements in later versions [Chapman et al., 2015; Clavijo et al., 2017; Alaux et al., 2018]). It was an extraordinarily welcome achievement for someone who had created the first kinds of wheat genome maps in the 1970s (Rimpau et al., 1978), deduced gross genome maps built upon methylation patterns in the early 1990s (e.g. Moore et al., 1993) and seen denser molecular marker wheat maps appearing from close colleagues through the 1980s, 1990s, and 2000s (Gale and Devos, 1998).

Any commentator reviewing progress in chromosome biology over the past couple of decades will recognize the vital roles of deep computing with all the associated open source software packages. Genome browsers have become necessary routine tools of the geneticist and chromosome analyst. The forces that created communal public databases have generated huge informational wealth.

## Whole chromosome DNA sequencing reveals organizational order

What have we learned from whole genome sequencing? Annotations of complete genome sequences have revealed essentially all the genes and their surrounding sequences, their position in each chromosome, the precise variation in all the repeat families, and the distribution of repeat members throughout the genome. The sequence of one or a few genomes per species provides only a static snapshot of the chromosomes of the species. It requires sequences from many carefully selected accessions to provide estimates of the diversity within a species, as was gained from the 1,001 Arabidopsis genome project (Alonso-Blanco et al., 2016) and the 3,000 rice genotypes (Wang et al., 2018; Carpentier et al. 2019). Herein lie some challenges for the future. The many monocot and dicot genomes now sequenced (Kersey, 2019) make it impossible in this perspective to cover all their salient features. However, below are a few of the general points on chromosome organization that have come from whole chromosome DNA sequencing and molecular biology. All teach us the outcomes of the forces that, on the one hand, generate variation during evolution and on the other preserve and exploit variation for species survival.

At the end of the 1970s, it was concluded that the variation in total DNA amounts between plant species was predominantly due to repeated sequences, disregarding polyploidy. This has been confirmed by the complete genome sequences (Michael, 2014). This means that in spite of the huge variation in DNA contents the number of genes in haploid genomes has been kept relatively constant. Our interspecies DNA hybridization studies in the 1970s led to the conclusion that cereal genomes were built from millions of short sequences, less than 1,000 bp long and with repeats being interspersed with short non-repeated sequences. Table 1, taken from (Mascher et al., 2017), shows the numbers and sizes of repeated elements scored from the whole genome sequence of barley (Mascher et al., 2017). It confirms the general conclusions of the 1970s (Smith et al., 1976; Rimpau et al., 1978).

**Table 1:** The repeated element annotation statistics in the barley genome

	% of genome	% of T. elements	Number	Number (%)	Size (Mb)	Average length (bp)
Element	80.8	100.0	3,408,238	100.0	3,695	1,084
Class 1: Retroelement	75.2	93.1	2,881,139	84.5	3,439	1,194
LTR Retrotransposon	75.0	92.7	2,859,922	83.9	3,427	1,198
Copia	16.0	19.8	588,579	17.3	732	1,243
Gypsy	21.3	26.3	765,584	22.5	972	1,270
Unclassified	37.7	46.6	1,505,759	44.2	1,723	1,140
None LTR retrotransposon	0.3	0.3	21,217	0.6	12	581
Line	0.3	0.3	19,173	0.6	12	605
Sine	0.0	0.0	2,044	0.1	1	355
Class 11: DNA Transposon Superfamily	5.3	6.5	473,797	13.9	241	509
DNA Transposon Superfamily	5.0	6.2	418,583	12.3	230	550
CACTA superfamily	4.7	5.9	375,421	11.0	217	578
hAT superfamily	0.01	0.01	607	0.0	0	402
Mutator superfamily	0.15	0.19	18,936	0.6	7	370
Tc1.mariner superfamily	0.02	0.03	8,199	0.2	1	134
PIF/Harbinger	0.08	0.10	9,007	0.3	4	402
Unclassified	0.03	0.03	6,413	0.2	1	191
Mites	0.20	0.25	52,112	1.5	9	178
Helitron	0.03	0.04	1,643	0.0	1	818
Unclassified	0.01	0.01	1,459	0.0	1	350
Unclassified element	0.32	0.40	53,302	1.6	15	274

Taken from Mascher et al. (2017).

**Figure 2:**
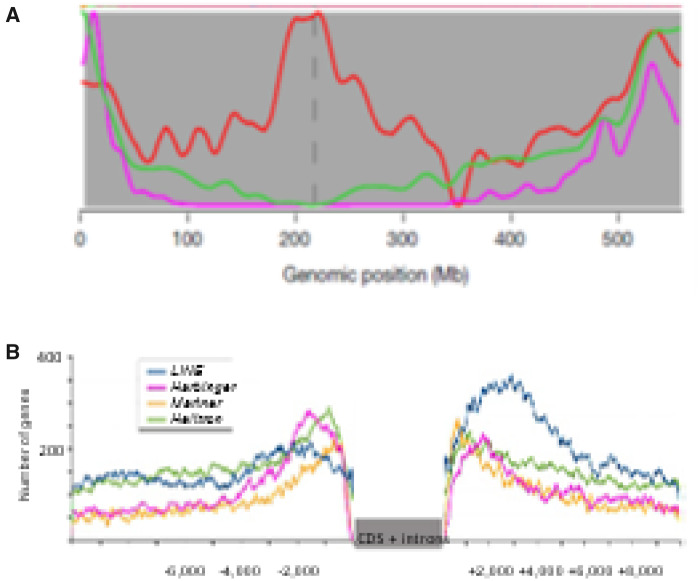
Organization of repeated sequences and genes along Gramineae chromosomes (taken from Mascher et al., 2017). The results are for the barley genome but are typical of other species’ genomes. A, The genes and recombination rates increase toward the end of the chromosomes. The GC content is skewed by the presence of higher GC repeats around the centromere and lower GC repeats in the middle of the arms. The centromere is marked by the dotted line. Green line = Genes, 2.1–29.3 per Mb; Purple = Recombination rate, 0–1.7 cM per Mb; Red = GC content (%) 43.9–45.0. **B**, The frequencies of genes possessing transposons at different distances upstream or downstream from the coding sequences. Helitron, Harbinger, and Mariner elements are commonly located close to where gene regulatory sequences are expected to lie.

In most plant species, genes are more concentrated toward the ends of chromosomes, as illustrated in Figure 2, A for barley, and these trends are more marked in species with larger chromosomes where the pericentromeric blocks of repeats containing only the occasional gene are much longer. Centromeres and telomeres are comprised of arrays of repeats, tandem arrays of short repeats but also complex combinations of LTR transposons, old and young (Simon et al., 2015). The tandem arrays appear to be functionally important for enabling centromeres and telomeres to perform their roles.

Patterns of recombination frequencies along chromosomes are similar to the distribution of genes, which is consistent with recombination occurring preferentially in/around genes (Figure 2, A). In Arabidopsis, recombination hotspots occur in promoters of genes enriched with CTT motifs, or an A-rich motif and where chromatin modifications include Histone 2A.Z and Histone 3K4me3 modifications, as well as CCN motifs within genes (Shilo et al., 2015). In maize, recombination hotspots also occur in promoters and transcription terminator sites where there are fewer nucleosomes and hypomethylation but show only limited association with H3K4me3 sites (He et al., 2017). There appears to be no correlation between rates of transcription and crossing over frequency (He et al., 2017). This co-incidence of recombination and genes undoubtedly reflects the value of recombination for generating genetic diversity, especially around the control regions of genes. It could also be that the order of genes along the chromosomes has been co-selected with recombination sites and frequencies to provide optimal genetic variation in plant populations. Transposons in euchromatin also can serve as recombination enhancers to play a role in shaping patterns of recombination, genetic diversity, and adaptation (Kent et al., 2017; Choi et al., 2018; Underwood and Choi, 2019). These sorts of discoveries have great significance for population biology.

Transposons inserted into or close to genes (see Figure 2, B) can and do create functional novelty (Hirsch and Springer, 2017). Such elements are shorter on average than those inserted in other regions and generally belong to smaller families. Helitrons, common kinds of autonomous or non-autonomous transposons believed to replicate by a rolling circle mechanism, and Harbinger elements, common elements with terminal inverted repeats and which produce a 3-bp duplication of the target site upon integration, have a clear preference for promoter regions in barley (Figure 2, B; Mascher et al., 2017). Elements close to genes frequently affect gene expression; they can provide novel promoters and have major influence on gene transcription by affecting local chromatin loop formation based on their methylation patterns (see the “Chromosomes have 3-D order and links to one another in nuclei” section). The amounts of transposon and other DNA between genes are generally related to genome size, i.e. there is less DNA separating genes in the small rice chromosomes than in the larger sorghum chromosomes, for example.

The lower overall density of transposable elements toward the distal regions where genes are concentrated may be the result of strong selection against disturbances there or the result of stochastic elimination by the higher rates of recombination (Dai et al., 2018). Assessments of the more recently arisen elements in wheat and *Aegilops tauschii* have led to the conclusion that initially they become distributed throughout a chromosome but over time become eliminated near the distal ends by recombination (purifying selection). This makes the older elements appear to be less concentrated distally. Also, there appears to be a positive relationship between genome size and recombination rate suggesting that purifying recombination is more active in genomes where repeats have a higher probability of being close to genes (Ross-Ibarra, 2007).

While numbers of transposons are lower nearer the ends of chromosomes, there appears to be greater diversity of types of transposable elements in distal relative to central regions, perhaps because specific members of families are sustained by selection. CACTA-type elements, which move by element excision, are overrepresented near the ends of chromosomes in some species, e.g. wheat (Daron et al., 2014), often in association with duplicated genes, especially where the duplicated gene sequences have been moved to locations different from the original copies. These elements are known to have the ability to capture pieces of genes and move copies elsewhere, as well as causing chromosome breaks and translocations during transposition (Daron et al., 2014).

Solo LTRs are common. These arise from unequal intra-chromosomal recombination between two LTRs of an intact element. Many truncated elements arising from non-homologous recombination exist. The over representation of solo LTRs, truncated LTR elements, and degenerated elements in proximal regions is further evidence for processes that lead to rapid elimination of whole elements in the distal segments where recombination is focused (Dai et al., 2018). There is much more to be learnt from genome structure because it reflects the outcomes of the dynamic between the mutagenicity of the perpetual generation and deletion of transposons and selection to sustain fitness (see the “Transcripts and small RNAs regulate chromosome activity through chromatin/epigenetic modifications” section).

## Numbers of genes, genetic maps, and gene synteny

The publication of the annotated Arabidopsis (2000) and rice chromosome sequences (2002) provided a window into the numbers of genes in plant species. Until then it was an open question, although EST libraries had provided good estimates. The question had originally been explored most successfully by Rot curves. Here, mRNA in excess hybridized to single/low copy DNA with kinetics that suggested tobacco had around 60,000 different expressed genes, i.e. similar to animals (Kamalay and Goldberg, 1980). This turned out to be reasonably accurate, given the nature of the approach. The numbers remained controversial however for some years. There now appear to be between 30,000 and 40,000 coding genes in plant chromosomes including many pseudogenes containing stop codons and frameshift mutations, tRNAs, miRNA sequences, and the rRNA 5S and 25S genes (Sterck et al., 2007; Michael and Jackson, 2013). However, it should be noted that any figure defining the numbers of genes in a genome may reflect more the history of the genome because there have been duplications of chromosomes in the ancestry of most plant species; it is likely that there might have been around 12,000–14,000 coding genes in the notional basic plant genome (Sterck et al., 2007). Also, there are splicing variants from many plant genes as well as multiple transcription start sites that could give rise to functionally variant products. Genome characterization today also includes the thousands of non-coding RNAs synthesized from intergenic, intronic, or coding regions (Liu et al., 2015), including the transcripts made by RNA polymerases IV and V (see the the “Transcripts and small RNAs regulate chromosome activity through chromatin/epigenetic modifications” section).

Geneticists need chromosome maps with markers that define variation within a species. The characterization of chromosomal DNAs and finding of nucleotide variants has had a wide impact on genetics in the last 30 years. In the early days of genetics, parents and progeny were distinguished by measuring phenotypes. Genetic linkage maps were assembled based on recombination frequencies emanating from meiotic crossovers. In the 1960s and 1970s, this could be achieved based on protein variants, isozymes, differing in size and/or charge using gel electrophoresis and later isoelectric focusing, as well as segregating traits. We had a large program to define and map the many seed storage proteins of wheat onto chromosome arms (Brown and Flavell, 1981; Brown et al., 1981). When restriction endonucleases became available it became possible to identify DNA-based polymorphisms after size-fractionated DNAs were transferred to a membrane (Southern Blot) and specific DNA sequences examined using radioactively labeled DNA or RNA probes, including for the wheat seed storage proteins (e.g. Thompson et al.,1983). Such restriction fragment length polymorphisms (RFLPs) revolutionized the making of multi-marker chromosome maps but had several technical limitations; they involved using radioactive isotopes, were relatively expensive, and did not easily scale to very large numbers of markers. A(amplified)FLPs were a very useful variant used in the construction of many plant chromosome maps as were Randomly Amplified Polymorphic DNAs (RAPDs). Variations in microsatellites were particularly useful markers. As DNA sequencing costs became lower, “genotyping-by-sequencing” from defined sites became very attractive to find polymorphic markers (Poland and Rife, 2012). We used this in Ceres, Inc. to generate a high-resolution linkage map of tetraploid *Miscanthus sinensis*, using genotyping-by-sequencing on heterozygous recombinants, identifying all 19 linkage groups for the first time (Ma et al., 2012). This was one of the most complex maps to have been generated without prior knowledge of the number of chromosomes.

Using RFLPs of genes as genome markers in the gramineae—e.g. rice, wheat, barley, sorghum (Sorghum bicolor), and maize, the order of genes along chromosomes was found during the 1990s to be conserved during evolution, reflecting the common evolutionary origin of the monocots more than 60 million years ago. My colleagues Mike Gale (Gale and Devos, 1998) and Graham Moore (Moore et al., 1995) were major players in this discovery. The synteny could be represented by taking chromosome blocks of each species and aligning them as concentric circles based on the order of the genes that they have in common. This gene synteny is of course in strong contrast with the major differences between species in the DNA sequences in intergenic and other regions. Is the conservation of gene order simply a leftover from the common evolution, or is it preserved for functional reasons? There is much more to learn before this question can be answered.

Especially noteworthy in chromosome alignments based on synteny is that mapped genes controlling important traits in one species point to where their homologs are likely to map in related species. The conserved synteny has enabled the molecular biology of large genomes, such as that of wheat, to become more tractable, based on the relative simplicity of the small genome of rice. For example, cloning of the Ph locus that regulates homoeologous chromosome pairing in wheat was aided by chromosome walking using the homologous rice genome segment as a guide, before the complete wheat genome sequence was available (Rey et al., 2017).

Today dense molecular marker maps exist for all the major crop species, with very large numbers of markers (many tens of thousands) for the major species, although not all the markers have been converted into the easy-to-use formats required in high-throughput plant breeding. They enable haplotypic segments to be followed through pedigrees and their breeding values linked to different environments and uses. This is a huge and commercially important achievement for plant breeding and crop improvement.

## Transcripts and small RNAs regulate chromosome activity through chromatin/epigenetic modifications

**Figure 3:**
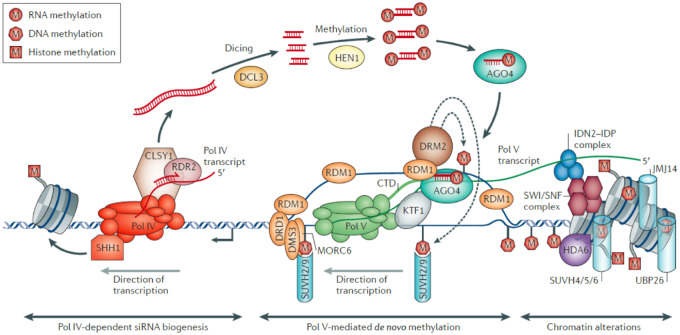
Establishment of post transcriptional silencing, RNA-directed methylation, and eventually stable transcriptional silencing of transposons. Left panel, Transcript of new active transposon by RNA Polymerase II is made double stranded by RNA-directed RNA Polymerase 6 and the double stranded RNA is then cleaved into 21 and 22 nt pieces by Dicers 2 and 4. These pieces become loaded on to Argonaute (AGO) 1 and guide cleavage of the RNA transcripts of the transposon to achieve post transcriptional silencing (PTGS) (Panda and Slotkin, 2013). Middle Panel, The 21–22 nt sRNAs can also trigger some methylation of the transposon by associating via base pairing with a Polymerase V transcript of the transposon. This complex attracts the DOMAINS REARRANGED METHYTRANSFERASE 2 (DRM2) which achieves some methylation of the transposon DNA and transcription gene silencing (TGS). Right panel, Polymerase IV can be attracted to the methylated transposon DNA and the transposon transcript can be made double stranded by RNA-dependent Polymerase 2 and cleaved with Dicer 3 into 24 nt pieces. Following incorporation into Argonaute 4 the 24 nt RNA can base pair with a Polymerase V transcript which stimulates DRM2 recruitment and dense methylation of the DNA. Once the transposon is substantially methylated then 24 nt RNAs are produced by the Polymerase IV pathway (Middle panel) to ensure continued dense methylation and transcriptional silencing. Reprinted from Matzke MA and Mosher RA, (2014) with permission from Nature/Springer.

The finding that a high proportion of the DNA in chromosomes is comprised of transposons made learning the mechanisms whereby they are kept quiet one of the highest priorities in chromosome research. Early in the 1990s mechanisms concerned with protecting genomes from viruses started to be uncovered, and since then a very large number of papers has revealed the overarching mechanisms in which transcripts and their small RNA derivatives keep repeated DNA essentially silent. In many ways these discoveries have been the most interesting of the 50 years because of their overarching implications. Who would have predicted that active forms of repeats are essential to keep them silent? The breakthrough came from recognizing the correlation between post transcription gene silencing (see the “Transgenes teach about transcriptional and post transcriptional silencing” section) and the presence of antisense short (25 nt) RNAs by my long-time colleague, David Baulcombe and his team (Hamilton and Baulcombe, 1999). In summary form (Figure 3, taken from Matzke and Mosher, 2014), when a new transposon enters the genome (by transposition or from another plant) its transcript is converted into double stranded RNA by RNA-dependent RNA polymerase 6 (RDR6; see left-hand side of Figure 3), and then two Dicer-like complexes cleave the RNA into double stranded 21–22 nt RNAs (Panda and Slotkin, 2013). These short single strands can base pair with more transposon transcripts followed by cleavage of the double stranded RNA to achieve post-transcriptional gene silencing (PTGS).

The 21–22 nt RNAs complexed with an Argonaute complex, AGO2, can also base pair with a different transcript from the transposon, that made by Polymerase V (see middle section of Figure 3) and these can attract the DOMAINS REARRANGED METHYTRANSFERASE 2 (DRM2) to methylate the transposon DNA. This results in a chromatin complex that is not transcribed, or barely so. Another polymerase, Polymerase IV, can be attracted to the methylated DNA and its transcript made double by RDR2. This can then be cleaved by Dicer-like 3 into 24 nt RNA pieces (see right-hand side of Figure 3). The association of the 24 nt RNAs with an AGO4 complex can interact with additional Polymerase V transcripts from the transposon and attract the DRM2 complex to achieve more extensive methylation of the transposon. Additional Polymerase IV transcripts from the more heavily methylated DNA can be made double stranded by RDR2 and when cleaved by Dicer-like 3 generate more 24 nt RNAs which can proliferate methylation of the complement of related repeats. Such highly methylated elements are typical of heterochromatin but this heterochromatin structure is maintained at each cell division immediately following DNA replication by the enzymes Met1, CMT3, and DDM1, not involving the 24 nt RNAs (Feng and Michaels, 2015).

Polymerases IV and V are recruited to transposable elements that already contain Histone 3K9me and cytosine methylation, respectively. Thus, the RNA-directed DNA methylation (middle and right hand side of Figure 3) is a self-reinforcing loop, which in the most extreme case can stimulate spreading of methylation and generation of a dense heterochromatic environment that restricts access to Polymerase II. This is highly desirable where transposable element activity needs to be silenced to avoid transposition. If elements escape such silencing then 21–22 nt RNAs (left and middle of Figure 3) can promote the post-transcription and then transcription silencing. When a transposable element close to a gene undergoes this methylation cycle, the resulting methylation can affect transcription factor binding within the gene promoter to positively or negatively affect Polymerase II transcription and gene expression (Zheng et al., 2012).

Polymerases IV and V clearly shape the epigenome by guiding methylation to specific genomic sites, among other things. While what determines the sites of Pol IV and Pol V transcription is still not fully understood, their products—the tens of thousands of non-coding RNAs—have now become recognized as major components of the eukaryotic transcriptome (Ariel et al., 2015; Liu et al., 2015). They are involved in a wide range of regulatory mechanisms impacting gene expression, including chromatin remodeling, modulation of alternative splicing, fine-tuning of miRNA activity, and the control of mRNA translation or accumulation. Their contribution to quantitative genetics remains to be uncovered. The extent of methylation in any particular gene locality is determined not only by the small RNA pathways but also by demethylation, catalyzed by glycosylases. It is therefore postulated that each region is monitored somehow and the right balance between methylation and non-methylation is optimized on a continuous basis in rheostat fashion, certainly after cell division (Williams et al., 2015).

The 21–24 nt siRNAs that silence or modulate a transposable element close to a gene could be from transcription of any member of the transposon family in the genome. Thus, perhaps a few active elements can facilitate the in trans silencing of large numbers of elements, in which case a cell needs to generate a balance between keeping all transposable elements in a family silent versus enabling selected ones to generate transcripts from which silencing siRNAs are created. Such complexities emphasize the necessity for co-evolution of all the features embedded in chromosomes to enable the right outcomes for gene control.

While particular patterns of DNA-based epigenetic marks correlate with and determine chromatin function they are coupled with histone modifications that also determine the establishment of the different chromatin structures. The discovery and characterization of histone variants has led to the realization that they play fundamental roles in the regulation of nearly every aspect of chromatin biology. For example, silent heterochromatin is marked by histone H3K9 (H3K9) dimethylation and H3K27 monomethylation. Histone H3K27me3 marks silent genes and genes with tissue-specific expression and is highly anti-correlated with gene body methylation. Histone 3K4 trimethylation marks actively transcribed regions. Disruptions in DNA methylation, H3K9me2 or H3K27me1, lead to chromatin decondensation and loss of transcriptional silencing (Feng and Michaels, 2015).

## The example of ribosomal DNA silencing by RNA-directed mechanisms

**Figure 4:**
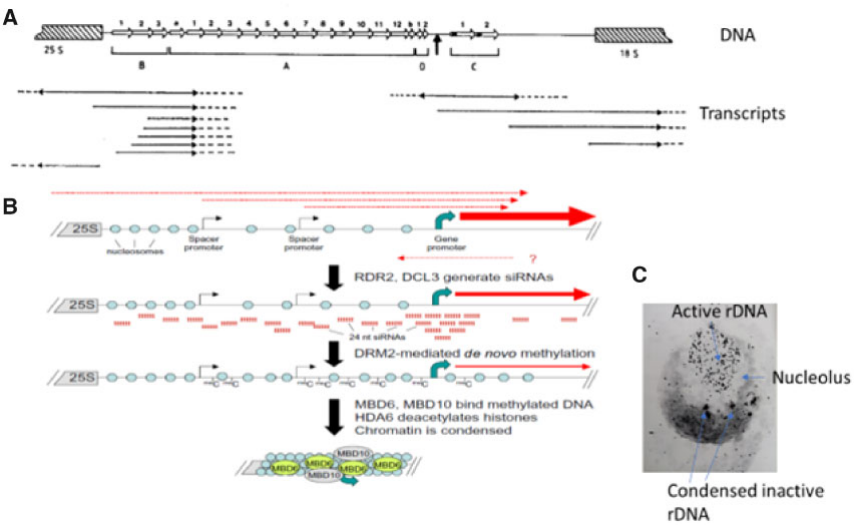
Control of condensation of multigene arrays of rDNA into heterochromatin. A, Map of rDNA repeat unit from wheat showing 12 135 bp repeats (labeled A) upstream of the major transcription start site (vertical thick arrow) and the intergenic transcripts initiated from different positions including readthrough from the 25S RNA sequence. Dotted lines indicate ends of the RNA transcripts not determined. DNA elements labeled B–D are other duplicated sequences. Results from Vincentz and Flavell (1989). Copyright held by ASPB. B, The organization of an rDNA repeat unit in Arabidopsis showing transcripts (red lines) from the intergenic promoters upstream of the major transcript (large blue arrow). The intergenic transcripts are processed into 24 nt RNAs that then direct do novo methylation of spacer sequences by domains rearranged methyltransferase 2. Binding of methylcytosine binding protein BD6 and MBD10 in conjunction with deacetylation facilitates chromatin compaction into heterochromatin. Reprinted from Preuss et al. (2008) with permission from Elsevier. C, In situ hybridization of labeled rRNA to wheat cells, showing the clustering of the silenced genes in heterochromatin and the active rDNA dispersed through the nucleolus (Flavell RB, unpublished data).

The major discoveries into the control of chromatin and gene activity in the last 20 years described above reveal how nature manages the hugely problematic issues emanating from the intrinsic generation of genome changes by transposable elements. They also aid interpretation of earlier studies on genes. During the 1970s I sought to add to the small body of knowledge on the control of gene expression by exploring the ribosomal RNA genes in wheat. These were chosen because in wheat there is a well-described example of nucleolar dominance in which one locus of thousands of rRNA genes is dominant or partially dominant over another and this could be scored cytologically in single interphase cells by observing the relative sizes of the nucleoli in the same cell. The differential expression of the nucleolar organizers that determines the relative extent of activity versus silencing is based on epistatic and “allelic” interactions and not solely on the numbers of rRNA genes at each locus. Silenced genes are condensed into heterochromatin (see Figure 4, C) while active genes are distributed throughout the nucleolus (Flavell et al., 1988) and nucleoli in the same nucleus frequently fuse. We subsequently showed that active gene units were less methylated than inactive gene units and also more susceptible to DNase 1 treatments due to not being in condensed chromatin (Thompson and Flavell, 1988). Thus, we were investigating a gene control system in which genes adopted different methylation and chromatin configurations in the presence of “paralogous clusters” as an outcome of the epistatic and “allelic” interactions.

Following leads of others in Xenopus and mouse, we sequenced the intergenic region of the repeated gene units encoding the 18 and 25S RNAs (Barker et al., 1988) and found reiterated 135 base pair repeats (suspected enhancers) located upstream of the main promoter of the transcript to which specific proteins bind and in which DNase 1 sensitive sites lie (Jackson and Flavell, 1992; Thompson and Flavell, 1988; Figure 4, A). Surveys of several nucleolar organizer regions (NORs) in different wheat lines suggested that dominance was correlated with the size of the intergenic spacer and the number of upstream 135 base pair repeats (Sardana et al., 1993). Transcription studies revealed not only transcripts from the main promoter but others reading through the 3′-end of the 25S gene into the intergenic region. In addition, but in much lower amounts, there were transcripts initiating in (three) repeated sequences upstream of the array of 135 bp repeats (see Figure 4, A; Vincentz and Flavell, 1989). We thus generated many observations on this interesting gene family but detailed mechanisms of control of the whole loci were unknown.

All these observations have now been pulled together with the model in Figure 4, B, from Craig Pikaard’s group, studying Arabidopsis (Mayer et al., 2006; Preuss et al., 2008). The silencing of genes into a heterochromatin configuration occurs by intergenic RNAs becoming double stranded in the nucleus and diced into 24 nucleotide RNAs by Dicer-like 3 as illustrated in Figures 3, 4. The 24 nt RNA/AGO complexes bring de novo cytosine methyltransferase2 to the site of the NOR and drive de novo methylation of the rDNA (Figure 3, right-hand side). This in turn attracts the methylcytosine binding proteins MBD6, MBD10, and histone deacetylase 6 (HDA6) to create a condensed chromatin state for the genes not required to be actively transcribed. The siRNAs do not silence the intergenic suspected upstream enhancers in genes available for transcription, perhaps because they are kept in an epigenetic state that allows continued transcription from the principal promoter. It is interesting with hindsight to note that in 1988 we saw the intergenic noncoding RNAs but had no idea that they would be the source of 24 nt RNAs that controlled methylation and condensation of rDNA. It took a reorientation of thinking and knowledge to generate the true picture.

The molecular explanation for the differential rDNA expression/dominance is still not fully defined, but it appears that the mechanism is at the level of the locus rather than the individual gene. Importantly, the dominance state is established in the first few divisions after fertilization of the egg cell (Neves et al., 1995). Thereafter, the dominance state is propagated throughout all the subsequent cell divisions of the life cycle, perhaps by the maintenance methylation systems which occur immediately after DNA replication and do not require an RNA transcript. Perhaps in the early zygote, NORs possessing genes with more upstream enhancers attract more factors, possibly including the long non-coding RNAs, which prevent their DNA methylation and conversion to heterochromatin and the gene arrays compete for such factors in the first few cell divisions after fertilization. It would then be the outcome of the competition that determines which arrays become condensed and inactive. Alternatively, it could be the sequences which flank the NORs that differentially influence the ability of NOR loops to become associated with a transcription factory in the young zygote (see the “Chromosomes have 3-D order and links to one another in nuclei” section).

## Chromosomal protection and epigenetic reprogramming between generations

The passage of chromosomes from one generation to the next without mutations, with the exception of the variation created by recombination, is vital. Keeping the transposable elements silent or from moving is especially important to preserve genetic integrity. However, the above example of the rDNA epigenetic changes in the early zygote illustrates that specific loci can undergo epigenetic reprogramming when in a new genetic environment and these can be stably inherited thereafter. Much epigenetic reprogramming of chromatin structure occurs during gamete formation associated with the major changes in cell biology unique to these development stages. Many details are being uncovered (see for example, Kawashima and Berger, 2014). In brief, during female meiosis DNA methylation decreases, including in transposable elements, while siRNAs build up in neighboring cells, and likely move into the meiocytes, to aid control of transcription and prevent transposable element transposition (Ibarra et al., 2012). Perhaps these epigenetic changes are connected with the chromosomal changes necessary for recombination. In pollen formation, the microspores also undergo loss of CHH methylation, transposable elements become active, and 21 nt RNAs accumulate, with the potential for aiding suppression of transposable elements when incorporated into the sperm cell (Calarco et al., 2012; Martínez et al., 2016). The reduced levels of methylation and histone marks in the gamete chromosomes become reinstated during the reprogramming of the chromosomal epigenetic features in the early zygote (Jullien et al., 2012), as noted in the rDNA example above. While new epigenetic variants can be generated during these phases and during reprogramming of gamete and zygote formation (and many authors have sought to correlate such changes with heterosis in hybrids), many of the histone marks and DNA methylation patterns are faithfully inherited from the parental chromosomes by the maintenance histone and DNA methylation systems that are deployed directly after DNA replication. Genome wide, there is therefore a mixture of faithful inheritance of parental epigenetic marks in histones and DNA and new epigenetic variation established during gamete and zygote formation. This new epigenetic variation can influence nuclear organization of chromosome segments as outlined in the “Chromosomes have 3-D order and links to one another in nuclei” section.

## Chromosomes have 3-D order and links to one another in nuclei

Our initial studies into repeated DNA sequences in the 1970s included those in constitutive heterochromatin (Bedbrook et al., 1980a; Jones and Flavell, 1982a). These centromeric and telomeric heterochromatin regions often fuse to form chromocenters. Our later investigations into rDNA (the “The example of ribosomal DNA silencing by RNA-directed mechanisms” section) also revealed that the rRNA genes that end up in heavily methylated heterochromatin structures fuse with each other. The localization of heterochromatin in interphase nuclei and its frequent fusion with other chromosomal regions carrying similar DNA sequences show that such regions provide, or impose, certain structural features on nuclei. This prompts many obvious questions about how chromosomes are organized with respect to one another and how genes within each chromosome are organized in the 3-D space of the nucleus and to each other. To fully understand chromosomes we need to understand how they are packed within nuclei and how the organization is influenced by environments, cell differentiation, and gene activity. Much new insight has been gained in the last 10 years, transforming the ignorance about chromatin organization in interphase nuclei that was present when I joined the Cytogenetics Department at the Plant Breeding Institute in 1969 and started observing the highly condensed metaphase chromosomes (see Figure 1, C).

**Figure 5:**
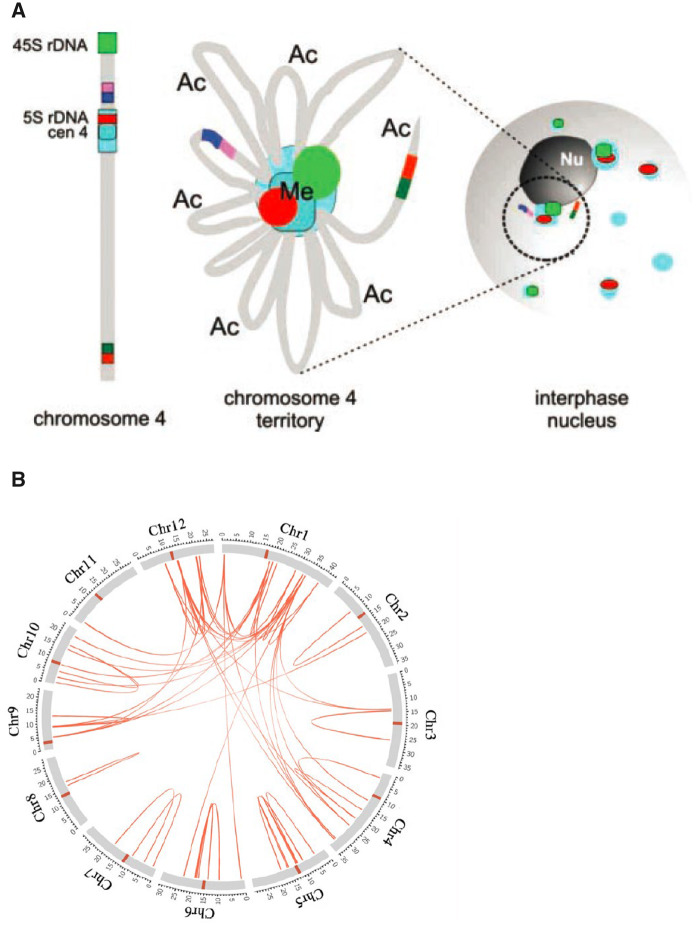
Linkages within and between chromosomes in interphase nuclei. A, Chromocenter-loop model for the organization of chromosome 4 of Arabidopsis. Heterochromatic regions, including 5S and rDNA, compartmentalize into one chromocenter with euchromatin loops around it. Me = highly methylated. Ac = enriched in acetylated histone H4. Taken from Fransz et al. (2002). In the interphase nucleus fewer than 10 centromeres are displayed indicating some fusion. B, Intra and inter chromosomal links in rice nuclei as revealed by HiC mapping. Reprinted from Dong et al. (2018). The centromeres are marked with the orange bars.

It has been recognized for over 100 years that in interphase cells of species with larger genomes, chromosomes are found in the “Rabl” configuration (Rabl, 1885) with centromeres and telomeres at opposite poles of the nucleus. This is the result of their positioning during the previous mitosis and cell division. Species with small genomes likely have a simpler nuclear organization than species with larger genomes where chromatin segments comprising genes and lots of repeated sequences are more frequent (Dong et al., 2018). Arabidopsis has short chromosomes arranged in a rosette configuration, such that the euchromatic chromosome arms, richer in acetylated histones and active genes, loop out from the fused chromocenters (Figure 5, A) that include the rDNA and nucleoli from chromosomes 2 and 4 (Fransz et al., 2002; Tiang et al., 2011). It is becoming accepted that each chromosome occupies a distinct territory in the nucleus but it remains unclear if the territories of different chromosomes have a fixed disposition with respect to others or their homologs. Many early studies on monocot species claimed that homologs were in closer contact with one another in nuclei (Flavell, 1985) but this has been contested. Fransz et al. (2002) reported that homologs appear to be associated in the same part of the nucleus in Arabidopsis. Moreover, a considerable number of nuclei displayed perfect alignment of homologous subregions, suggesting physical trans-interactions between the homologs (Fransz et al., 2002). Studies on other species come to different conclusions. For example, in the barley nucleus with its Rabl structure homologs show no preferential association (Mascher et al., 2017). In hexaploid wheat there is some separation between the constituent subgenomes and there is higher frequency of interactions between homoeologs than non-homoeologs (Concia et al., 2020). Certain rice chromosomes appear to lie closer together more often than expected if their disposition was random (Dong et al., 2018). In a study of interspecies cereal hybrids, parental genomes lay in various non-intermixed configurations, including lateral and concentric arrangements, leading to the conclusion that haploid sets were spatially separated throughout the cell cycle (Heslop-Harrison and Bennett, 1984; Flavell, 1985). This may be because each chromosome lacked a true homolog. Perhaps what matters more than how whole chromosomes are arranged is how specific regions of chromatin are linked together, or not, at the right time in the right way to facilitate cell function.

Hi-C technology, in which DNA sequences close to one another in a nucleus are co-isolated and identified by DNA sequencing, has opened up in the last 10 years (Figure 1) the means of assessing the spatial relationships of short segments of DNA/chromatin to one another (Feng et al., 2014; Liu et al, 2016; Mascher et al., 2017; Dong et al., 2018; Concia et al., 2020) and been deployed on a range of plant species (Dong et al., 2018). This has taken the dissection of nuclear and gene architecture into new territories. Strong interactions between the large chromocenters were readily seen, as were associations between telomeres. In addition, specific interstitial heterochromatin regions within and between chromosomes have been found to be extensive (Feng et al., 2014; see Figure 5, B for rice). They can link with the pericentromeric chromatin too. Such long-range interactions linking short heterochromatic regions and correlating with H327me3 or H3K9me2 marks provide much structure to the nucleus and implies that they are major forces shaping the overall positioning of chromosomes. In consequence they must also anchor the large loops of chromatin that are joined to these heterochromatic islands, as seen by (Fransz et al., 2002; see Figure 5, A). It has also been speculated that the interactions between the islands may aid in sustaining the silencing of the transposable elements and highly repeated sequences that lie within them by sharing the epigenetic machinery that results in them attaining similar chromatin states (Dong et al., 2018).

In studies of Arabidopsis nuclei at resolutions closer to the sizes of gene and gene clusters, over 1,000 kbp-sized segments of chromatin bounded by insulator elements have been uncovered. These segments contain active genes and have much higher intrachromosome interactions than to neighboring regions which are enriched in repressive epigenetic histone marks H3K27me3, H3.1, and H3.3 (Liu et al., 2016). The intrachromosome interactions are likely to involve loops where the bringing together of (distantly) linked chromatin segments is part of the gene expression control system. Many genes are held in single gene loop configurations formed from contact between their 5′- and 3′-ends. These are more frequent for highly expressed genes but can occur for silent or low expressed genes too. Similar conclusions have been drawn from detailed studies on hexaploid wheat (Concia et al., 2020). Specific intrachromosomal and interchromosomal associations were found. Folded chromatin regions (insulator elements), richer in methylation and silencing epigenetic marks and transposable elements, could be distinguished from gene-rich boundaries (loops) having epigenetic marks more typical of active gene structures. Gene–gene loops occurred more frequently between genes having similar expression levels and epigenetic marks, implying a correlation between active gene pairs and their 3-D spatial proximity. These loops were also foci of RNA polymerase II, suggesting that they are the sites of transcription clusters or factories (see Figure 1, C; Concia et al., 2020). All these studies and the clustering of specific alleles in nuclei as seen for the flowering time gene FLC (Rosa et al., 2013) suggest that functionally related genes are not randomly arranged in 3D and the positioning of genes within the nucleus is intimately linked with their related expression and functions.

Whatever specific chromatin structures occur they are likely to vary with changes in gene transcription and cell differentiation. Noteworthy is the extensive reduction in chromocenters in Arabidopsis prior to bolting that is followed by a recovery of the heterochromatin domains after elongation of the floral stem. This transient reduction in chromocenters is associated with decondensation of chromatin in gene-rich regions (Tessadori et al., 2007). Such substantial chromatin remodeling and changes in nuclear architecture during the floral transition reinforce the necessity to study chromatin and nuclear architecture in many cell types and under different growth conditions.

It is now clear that local epigenetic histone and DNA modifications, gene density, transcriptional activity, recombination, replication origins, and cell division all interact to influence the local packing of chromatin segments and hence 3-D organization of chromosomes and micro-segments of chromosomes in nuclei. The key roles that chromatin structure play imply that species with different genome sizes and distributions of genes and repeated sequence families may display variant gross 3-D organizations but these are based on underlying mechanistically common principles. It is also likely that the concentration of genes nearer the ends of chromosomes, the order of genes along chromosomes, and the accumulation of repeated sequences nearer the centromeres have been selected to enable chromosomes to function and interact locally more efficiently in the 3-D space of the nuclei. The linking of nuclear architecture to networks of gene/non-coding RNA expression and vice versa is destined to provide essential information on how cells function.

## Modifying chromosome information and plant traits by inserting novel genes and gene editing

Molecular geneticists and plant breeders have long dreamed of being able to modify existing alleles or insert new genes, specially designed, into the chromosomes of their favorite plant species to evaluate gene function, to provide new highly desired traits, for example disease resistances, and also to avoid backcrossing new alleles/genes into a given genotype. The value of this was obvious to me from the time I entered the plant breeding world after my postdoctoral fellowship. I therefore took special note of the most powerful and remarkable of discoveries around the late 1970s that crown gall disease of plants was the outcome of the transfer of a piece of DNA (T-DNA) from a plasmid (the Ti, tumor-inducing plasmid) of *Agrobacterium tumefaciens* into dicot cells and incorporation of the DNA into plant chromosomes (Nestor, 2015). This outstanding and consequential piece of both bacterial genetics and plant biology changed the course of plant science. It opened up in 1982/3 the means of moving genes from bacterial cloning vectors into plants via infecting plant tissue cultures with the modified bacteria, selecting in culture the cells receiving the DNA followed by regenerating the selected cells into whole plants using regimes involving switches of hormones. The initial achievements of transformation and selection of genetically transformed cells were achieved essentially simultaneously by four groups, including our own (Bevan et al., 1983; Fraley et al., 1983; Herrera-Estrella et al., 1983), partly in collaboration and partly in competition with one another.

The Ti plasmid is a large and unwieldy plasmid and so a binary system was devised enabling one’s favorite gene to be inserted easily into a small plasmid and other necessary transacting functions being supplied by the large Ti plasmid. Various plasmid vectors for plant genetic engineering were devised within our group by Michael Bevan (Bevan, 1984), and these pBI vectors were distributed widely to help boost the adoption of the technology worldwide. Later Richard Jefferson joined the team and he and Mike Bevan designed a series of vectors that contained the “GUS” reporter coding sequence that enabled cells carrying the transgene to be identified and the level of expression of the “GUS” genes quantitatively assessed by measuring the conversion of glucuronamides to other forms (Jefferson et al., 1987). These vectors were also widely distributed and had a large impact on the field, because they facilitated the identification and characterization of putative promoters inserted to drive the GUS gene. Other reporter genes, such as GFP variants, have also been extremely valuable in assessing chromosomal gene activity in transgenic plants.

Later, our team in the company Ceres, Inc. and others found that *A. tumefaciens* inserts T-DNA preferentially into promoter and 3′-regions of genes—places we would now describe as having open chromatin (Schneeberger et al., 2005), but when active T-DNA gene expression is not selected then the integration sites are much more random (Kim and Gelvin, 2007). Many details of how the T-DNA is transferred into a double strand break of the plant chromosome have been discovered (Nestor, 2015; Gelvin, 2017). From the early days of transformation, rearrangements of T-DNA and neighboring sequences were detected. We now know from whole genome sequencing of transgenic lines that T-DNA insertion commonly leads to (i) insertions from both sides of the double strand break, (ii) local rearrangements and deletions elsewhere where T-DNA became inserted and then was lost, and (iii) epigenetic changes within and around the T-DNA insertions (Gelvin, 2017; Jupe et al., 2019). The epigenetic modifications frequently lead to silencing of the T-DNAs. There are many implications from these various outcomes of T-DNA insertions. Not least is the need to find transformants in which the inserted genes are completely stable and single copy for commercial applications—something more challenging for the large crop genomes that are more susceptible to epigenetic modification and silencing. DNA has also routinely been inserted into plant cells and the cells regenerated into whole plants by attaching the DNA on to (gold) particles and shooting them via a Gene-gun (Altpeter et al., 2005). This approach can give rise to single inserts but also frequently multigene insertions, often at a single locus.

Some laboratories explored the use of matrix attachment regions to serve as genetic insulators to prevent T-DNA inserts from affecting and being affected by neighboring chromosomal sequences (Singer et al., 2011). Many saw the value of being able to target new genes into specific positions in the genome, not at random. Systems to achieve site-specific integration have been developed. One of the most successful was based on the Cre recombinase from bacteriophage lambda which provides the ability to insert DNA at pre-existing lox sites. Rounds of such transformations enable multiple genes to be stacked at the same chromosomal site so that the traits are always co-inherited (Ow, 2011; Birchler, 2015). Another approach was to generate double strand breaks at specific chromosomal sites by Talens, Zinc finger, or CRISPR/Cas site-specific nucleases, thereby enticing the introduction of T-DNA into the cut sites. Gemini viruses have also been used to introduce the DNA at double strand breaks (Dahan-Meir et al., 2018). All these approaches are relevant to the creation of synthetic chromosomes, based on, for example, minichromosome engineering (Birchler, 2015).

In recent years, gene editing has emerged and become one of the most exciting additions to the arsenal of the plant geneticist and plant breeder because it enables heritable variation to be designed and created in targeted ways in elite genotypes, thereby avoiding backcrossing (Luo et al., 2016; Schmidt et al., 2019). I was therefore thrilled to serve recently for a period as Chief Scientist of a new company in which genome editing is a major feature. The extraordinary discovery of gene editing from the bacterial world exploits the means of using the CRISPR/Cas and similar nucleases to achieve double strand breaks at sites defined by guide RNAs (e.g. Gil-Humanes et al., 2017). Repair of the breaks via homologous recombination or non-homologous end-joining frequently creates mutations. It is relatively simple to achieve targeted deletion of bases but more difficult to create several specific base changes within a gene to create a different protein, for example, or within a promoter to modify gene expression. But progress is moving rapidly. Researchers are now making edits to many selected genes in one step by targeting double strand breaks at the loci (Ma et al., 2015). Perfection of this will make the creation of multiple design changes without the need to backcross even more valuable. Discovery of the means of localizing proteins, such as the CRISP/Cas nuclease to specific sites by guide RNAs, is being exploited to tether other proteins, such as methylases, to achieve methylation of specific DNA sequences. Thus, it appears that many chromosomal changes may be developed in the future via targeted protocols.

Editing protocols require introduction into plant cells of the molecules that make the edits and then regeneration of whole plants from the edited cells. They thus rely on transformation systems such as that provided by *A. tumefaciens*. These are not efficient enough to produce specifically edited plants without selection of the edited cells. Therefore, some scientists are first making transgenic lines carrying highly expressed nuclease, introducing guide RNAs for the genes to be modified into such lines and then selecting progeny containing the desired edits which are found to occur at a higher frequency. The transgene can be deleted by crossing it out in later generations.

There remains the major question of what changes need to be made to chromosomes to improve specific plant varieties using T-DNA insertions and genome editing. We need to know much more about the genetic basis of trait improvement in our key crops to put genome editing to best use for crop improvement, but many papers are emerging with useful beginnings. Another exciting use of genome editing is to create novel variation that breeders have never been able to evaluate. This has enormous potential and value. Nice illustrations of this have been provided in tomato where editing of upstream promoter regions has created a range of variants in the same genetic background from which breeders can choose what is most useful (Rodríguez-Leal et al., 2017). The next generation of geneticists and plant breeders has much to look forward to.

## Transgenes teach about transcriptional and post transcriptional silencing

All who create transgenic plants, particularly in species with larger genomes, know that a characteristic and initially disturbing feature of transgenic plants transformed with the same vector is the variable level of expression of the transgene, apparently linked to its chromosomal position. Thus, there is an epigenetic basis to the expression (see the “Modifying chromosome information and plant traits by inserting novel genes and gene editing” section). This was an important observation not only because of the practical issue of what expression level to conclude is characteristic of the gene construct but also because it implied that levels of expression were being determined by the surrounding sequences or, more accurately, chromatin structure of either the construct, the surrounding chromosome environment or by mRNA degradation. It was also found frequently that transgenes become silent in later generations, implying that chromosomal epigenetic states change between the generations (see the “Chromosomal protection and epigenetic reprogramming between generations” section). A whole field of research into gene silencing developed, transcriptional and post transcriptional, because of the importance of being able to create transgenic plants that had well-defined properties and that were completely stable across many generations. The research turned out to be extraordinarily significant because it helped stimulate the discoveries of the control of chromosome activities by small RNAs described above in the “Transcripts and small RNAs regulate chromosome activity through chromatin/epigenetic modifications” section.

**Figure 6:**
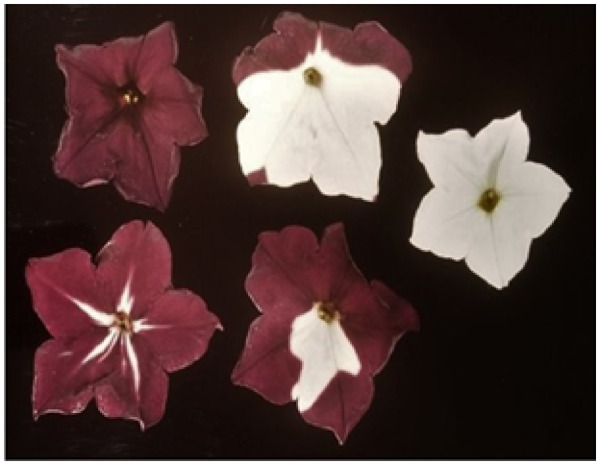
Patterns of gene silencing in petunia petals carrying various T-DNA structures each comprising petunia chalcone synthase cDNA driven by CaMV 35S promoter and Nos terminator (Cluster et al., 1996). Purple areas display parental pigments while white areas lacking anthocyanins have mRNAs from both transgenes and endogenous genes degraded. All transgenic plants have a variety of chalcone synthase RNA transcripts, influenced by the structures of the multiple T-DNAs. The flower top left is from a parental non-transgenic plant. Plants with all white flowers often produce only white flowers, while those with white and purple sectors show variation in the extent of patterning. The patterns of silencing reveal how the developmental biology of the flower interacts with RNA processing pathways to create the silencing siRNAs. Movement of siRNAs from cell to cell may also be involved (see Metzlaff et al., 1997, 2000; Que et al., 1997; Cluster et al., 1996; Flavell et al., 1999).

One of the first examples of post transcriptional silencing, described as “cosuppression” was the loss of chalcone synthase in petals of different transgenic petunia plants first created in Advanced Genetic Sciences in Berkeley, CA (Napoli et al., 1990). At the time, the silencing of both added transgenes encoding chalcone synthase and the endogenous chalcone synthase genes in petals was unexpected, mystifying and appeared to undermine the purpose of adding transgenes, which was to enhance the levels of chalcone synthase (see Figure 6). This seemed important, so my group explored the petunia plants further to find out why and how transgenes silenced themselves and endogenous copies. We published a series of papers (Metzlaff et al., 1997; Flavell et al., 1999; Metzlaff et al., 2000) proposing the formation of double stranded RNA and cleavage by a RNase specific for double stranded RNA as the mechanism for post transcription degradation of the mRNA (PTGS; Metzlaff et al., 1997), and this appeared to be associated with epigenetic changes in the chromosomal DNA and multicopy transgenes/inserts (Cluster et al.,1996; Flavell et al., 1999). We failed to look for double stranded RNAs as small as 21–24 nt (see Figures 3, 4). We did see, however, many nuclear RNAs that were truncated in different positions and polyadenylated or not, with some being unspliced or spliced in aberrant positions in tissues displaying post transcriptional silencing (Metzlaff et al., 2000). These developmentally regulated, unusual RNAs correlated with messenger RNA turnover in non-transgenic as well as transgenic plants but were present in much greater amounts in transgenic plants. In hindsight, what was happening was the synthesis and generation of nuclear non-mRNA species, formation of double stranded RNAs, and their cleavage to form specific, small 21 nucleotide sense and antisense RNAs from the larger RNAs by the complex pathways described in the “Transcripts and small RNAs regulate chromosome activity through chromatin/epigenetic modifications” section and Figure 3 (Luo and Chen, 2007; De Paoli et al., 2009; Wroblewski et al., 2014). The methylation of the chalcone synthase genes in cells undergoing post transcriptional silencing that we observed may reflect the activities of the 21 nt RNAs in stimulating methylation and transcriptional silencing as also shown in Figure 3. The unspliced RNAs we detected are likely to be the Polymerase IV or Polymerase V transcripts arising from appropriately methylated genes. For this perspective on chromosomes, it is relevant to recognize that the activity, number, structure, and arrangement of the transgenes determine the induction of the RNA synthesis and processing systems responsible for post transcription and transcriptional silencing, as well as the phenotypic outcomes (Cluster et al., 1996; Que et al., 1997). No doubt these factors are relevant to all genes and subject to natural selection. They teach us some design rules for how to make synthetic changes to chromosomes or select the desired modifications.

Analyses of the pigment patterns of these petunia plant flowers revealed that (i) epigenetic switches occur frequently in or close to meristems and between sexual generations, which suggest that this is when epigenetic switches frequently occur in chromosomes (see the “Chromosomal protection and epigenetic reprogramming between generations” section) and (ii) that small RNAs may move through meristems and between cells to affect the epigenetics and RNA biology of recipient cells (Que et al., 1998; Kobayashi and Zambryski, 2007).

The development of the field of transcriptional and post transcriptional silencing emanating from a huge number of subsequent studies has radically changed our thinking about the control of chromatin structure and activities, including gene expression as well as the design of transgenes and transgenic chromosome structures for research and applied use (Wroblewski et al., 2014; Jupe et al., 2019)*.* Understanding more fully how chromatin structure and neighboring sequences can influence transgene activity, due to DNA and histone methylation, non-coding RNA transcription, siRNAs, and antisense RNAs tells us that genetic engineering, especially in crops with larger genomes, is more complex than we thought 20 years ago and demands greater awareness of up-to-date chromatin and chromosome design rules. Natural selection has built in mechanisms to minimize the effects of foreign sequences. As would-be genetic engineers we have to be alert to these mechanisms. We also must continue to worry about rearrangements from T-DNAs and other co-incident changes that can accompany the insertion of transgenes.

## Concluding perspective on the 50-year adventure

During the last 50 years, plant chromosome research has gone from being cytology and cytogenetic based, where essentially nothing was known about the DNA within any plant chromosome, to where fully assembled genome sequences and transcriptomes are being generated almost routinely and 3-D organizations of nuclei are becoming understood, even for large genomes such as that of hexaploid wheat (Figure 1, C). We now recognize that the functional features of chromosomes need to be seen through the lens of how local chromatin structures are regulated, maintained, and inherited. This integrated, but still evolving, picture emphasizes that 3-D multimolecular structures underpin informational readouts and all chromosomal activities. Huge numbers of genetically modified plants have been created carrying redesigned genes specifically created to test hypotheses and give plants new properties. Thus, most of the academic goals in mind in the 1970s and much more have been achieved.

### The big breakthroughs

Looking back over the 50 years, what major developments have been responsible for the progress? I would nominate the following six major topics: (1) the recognition in the 1960s and 1970s that most of the DNA in plant genomes is comprised of repeated sequences and not genes, with all the ramifications that this brought in terms of genome stability, genetic load, sequence turnover, potential sources of genetic variation, and the need to keep most of them silent. (2) The extraordinary technical developments in DNA cloning, followed by DNA and RNA sequencing with their reductions in cost together with all the bioinformatics to interpret the outputs from the different sequencing approaches. (3) The choice of Arabidopsis and rice as the major genetic models for plant biology and the massive investment in their genetics to link chromosome biology with cellular and whole plant traits. These species have dominated discovery since the late 1980s. (4) The ability to modify chromosomes by inserting new genetic information as transgenes. (5) The largely unforeseen discoveries revealing that DNA and chromatin epigenetic modulations are programmed by small RNAs created by complex processing pathways from longer transcripts. (6) Genome wide molecular and genetic studies that have revealed the patterns, locations, and controls of recombination that show how crossovers do not occur at random (as known also by the early cytologists) but are localized in or near genes. I have left out genome editing simply because its value is in its infancy, although it promises to be of massive value, for research and application.

Most of these game-changing advances in plant chromosome research originated not from within plant science per se, but from advances outside the plant kingdom and investments made elsewhere across the life sciences, especially in the biotech industries who invented the tools that would later be used by us plant scientists, with the possible exception of *A. tumefaciens* transformation technology and even here bacterial genetics and DNA cloning were essential pre-plant discoveries. The crucial frameworks of novel thinking also came predominantly from outside plant science and imported into it by leaders who were intolerant of the status quo and bold and eager to start new approaches with new technologies. They are the ones who have moved the subject into new perspectives. They carried me along and brought all of us a long way toward achieving the practical dreams, and more, of 50 years ago, but then 50 years is a long time and huge resources have been spent.

### Who has benefitted?

What have 50 years of plant chromosome research done for crop improvement, given that was part of the driving dream in the 1970s? They have obviously created a new intellectual basis for plant improvement as well as a new vision and sets of tools and technologies for achieving the goals. The new vision comes from understanding the structures of chromosomes, the positioning of genes within them, and knowledge of some combinations of genes that underlie phenotypes/traits. Yields of the major crops have increased through more efficient plant breeding, and chromosomal polymorphic markers have become an obvious addition to plant breeding with high impact. However, they took a very long time to become widely adopted, because (i) the costs remained too high, (ii) most senior breeders were slow to accept the innovations, and (iii) the information of which markers are linked to which complex traits has been slow to emerge. However, genomic selection is now emerging as a useful outcome of chromosome-trait mapping, as traits become linked to combinations of markers. This brings hope for managing the genetic complexities of phenotypes and making plant breeding more efficient and effective. However, there is still much to be done to establish the haplotype segment/traits combinations to optimize crop varieties/hybrids for all the environments of the world where production is important. Furthermore, to deploy the technologies in commercial plant breeding programs, the technical systems must be fast, cheap, and reliable. Much more translational research and reductions-to-practice are needed to extract commercial value from the wealth of new knowledge. Who will do this? This is a major concern for the coming years.

What have 50 years of chromosome research done for societies? Sadly, we cannot yet say that our vision of the early 1970s for massive improvements in world agriculture through plant science innovations has emerged, with the exceptions of the transgenes conferring viral, herbicide, and insect resistances to crops. The latter were wonderful successes bringing benefits to several crops and many societies in terms of yields, safety, profits, and healthier environments. Other examples do exist but in contrast to the vast numbers of trait changes created via transgenesis in Arabidopsis and rice, commercial crop yield increases have been much more difficult to demonstrate via plant transgenes because (i) few scientists work with elite germplasm and carry out the tests in fields necessary to show a crop improvement, (ii) the genetics of most traits is complex, (iii) transgenes are often unstable across generations, and (iv) the costs of commercialization have been too high to warrant the research. All these have interfered with the natural flow of proven, relevant discoveries from plant science to agriculture. However, the overarching influence has been the fact that deployments of plant transgenes in agriculture have been almost completely undermined by pressure groups and then public opinion. This is a huge tragedy for plant science, investment into plant chromosome research, agriculture, and a massive loss for all those who expected benefits to accrue in agriculture from breakthroughs in plant chromosomal research. This major rejection of scientist-altered chromosomes raised serious questions about trust in plant science, scientists, and agriculture.

### The future

Progress in plant science will continue to depend on reductions to practice and in cost of all forms of genomics, bioinformatics, artificial intelligence, complex genome editing, rewriting of genomes, and real-time dynamics in cell biology of model and crop species. Plant chromosome biology will therefore likely rely on research in other non-plant model species for opening up new vistas and technologies, as has been so powerfully illustrated recently by genome editing. If so, then plant scientists and funding agencies would do well to follow the frontiers and predictions in, for example, human genetics and non-plant model species such as yeast and bacteria, to envisage the future frontiers of plant science and plant chromosome biology.

The massive discovery platforms that have been unleashed in rice and Arabidopsis must continue to drive opportunity but there is urgent need to find and commercialize big gains in elite lines of crops. That part of the big vision of the 1970s remains largely unfulfilled today.

Papers are now being published in significant numbers illustrating the ease and means of editing plant genomes. Therefore, we can look forward to an extraordinary proliferation of chromosome modifications, based on genome sequence knowledge, that will teach us much more about the finer points of chromosome architecture and function. It will take some time perhaps to achieve the more complex and multiple sorts of edits that will be needed, but non-plant models and improvements in the editing tools will show the way. Undoubtedly, we can gain the means of making radical changes to the crops that feed us, but whether societies will come with us is another matter. What is needed above all is to gain the confidence and appropriate legislation in societies around the world to enable significantly improved crop chromosomes modified by scientists to enrich agriculture and improve sources of food, feed, fiber, and energy. Only in this framework will progress in plant chromosome biology be recognized as a society-cherished activity providing essential services for the planet and its peoples’ welfare.

## Notes

The author responsible for distribution of materials integral to the findings presented in this article in accordance with the policy described in the Instructions for Authors (https://academic.oup.com/plphys) is: Richard B. Flavell (richflav@msn.com).
